# Aberrant reduction of telomere repetitive sequences in plasma cell-free DNA for early breast cancer detection

**DOI:** 10.18632/oncotarget.5083

**Published:** 2015-08-24

**Authors:** Xi Wu, Hiromi Tanaka

**Affiliations:** ^1^ Department of Medical and Molecular Genetics, Indiana University School of Medicine, Indianapolis, IN, USA

**Keywords:** telomere, plasma cell-free DNA, breast cancer, early cancer detection, real-time qPCR

## Abstract

Excessive telomere shortening is observed in breast cancer lesions when compared to adjacent non-cancerous tissues, suggesting that telomere length may represent a key biomarker for early cancer detection. Because tumor-derived, cell-free DNA (cfDNA) is often released from cancer cells and circulates in the bloodstream, we hypothesized that breast cancer development is associated with changes in the amount of telomeric cfDNA that can be detected in the plasma. To test this hypothesis, we devised a novel, highly sensitive and specific quantitative PCR (qPCR) assay, termed telomeric cfDNA qPCR, to quantify plasma telomeric cfDNA levels. Indeed, the internal reference primers of our design correctly reflected input cfDNA amount (R^2^ = 0.910, *P* = 7.82 × 10^−52^), implying accuracy of this assay. We found that plasma telomeric cfDNA levels decreased with age in healthy individuals (*n* = 42, R^2^ = 0.094, *P* = 0.048), suggesting that cfDNA is likely derived from somatic cells in which telomere length shortens with increasing age. Our results also showed a significant decrease in telomeric cfDNA level from breast cancer patients with no prior treatment (*n* = 47), compared to control individuals (*n* = 42) (*P* = 4.06 × 10^−8^). The sensitivity and specificity for the telomeric cfDNA qPCR assay was 91.49% and 76.19%, respectively. Furthermore, the telomeric cfDNA level distinguished even the Ductal Carcinoma *In Situ* (DCIS) group (*n = 7*) from the healthy group (*n* = 42) (*P* = 1.51 × 10^−3^). Taken together, decreasing plasma telomeric cfDNA levels could be an informative genetic biomarker for early breast cancer detection.

## INTRODUCTION

Telomeres are protective DNA structures that are located at the end of chromosomes, and proper telomere maintenance is indispensable for chromosomal integrity and overall genomic stability. Telomere maintenance is normally controlled by telomerase activity as well as telomerase-associated factors throughout the cell cycle in tissue- and cell type-specific manners [1–3]. It is well known that telomere length in white blood cells is inversely correlated with age, implying that telomere length may serve as a biological clock to determine the lifespan of a cell and an organism [4]. When telomere maintenance is disrupted by excessive erosion of telomeric DNA or loss of telomere binding protein function, the cellular DNA damage response (DDR) becomes activated to repair the dysfunctional telomere [5]. Persistent DDR induces a permanent proliferation arrest known as replicative senescence, which is thought to function as a tumor suppressor [6–8]. However, dysregulation of the DDR pathway allows cells to proliferate beyond senescence limits. When the cells reach a stage of persistent telomere dysfunction (termed telomere crisis), these aberrant processes lead to telomere fusions causing genomic instability via breakage-fusion-bridge (BFB) cycles. As a result, the ongoing genomic instability could increase the occurrence of further genetic or epigenetic alterations that would favor neoplastic transformation. Therefore, telomere crisis, characterized by extensive telomere erosion, chromosomal fusion, and genomic rearrangements, is an important early event in cancer development [9–11].

Initially, the mean telomere length was measured using Southern blotting, and demonstrated that the majority of invasive breast carcinomas displayed shorter telomeres than adjacent, benign breast tissues [12–14]. Additionally, results from *in situ* telomere length assessment (qFISH) indicated that modest telomere shortening occurred at the hyperplasia stage and a more significant shortening became prevalent as early as in Ductal Carcinoma *In Situ* (DCIS) [9, 15]. Telomere crisis has also been reported in human mammary epithelial cell *in vitro* culture models. For example, late-passage human mammary epithelial cells escape a stress-associated senescence-like barrier and acquire chromosome aberrations, including telomere fusions and translocations [10, 16]. Moreover, our group has recently demonstrated that telomere fusions are indeed present in early-stage breast tumors including DCIS [17]. Together, these findings provide strong evidence for the occurrence of telomere crisis-initiated genomic instability during early breast cancer development, probably at the transition from hyperplasia to DCIS [9, 15].

Mounting evidence indicates that extracellular, free-nucleic acids are released from the tumor cells and circulate in the bloodstream of cancer patients as tumor-derived cell-free DNAs (cfDNAs), along with normal cell-derived cfDNAs. The tumor-derived circulating cfDNA in plasma constitutes a potential source of genetic material for the identification of tumor-associated alterations, such as microsatellite instability, loss of heterozygosity (LOH), gene mutations, copy-number alternations (CNAs) and methylation [18–22]. Various methods have been established for the measurement of circulating cfDNA and the clinical utility of cfDNA assays has attracted significant attention as potential cancer biomarkers [23, 24]. Most of the current cfDNA assays require information on specific genetic or epigenetic alterations present in the original tumor lesion, therefore are limited to monitoring cancer progression. Alternatively, other cfDNA assays use a panel of frequently mutated genes, although the detection sensitivity still remains unsolved. There are thus far no established assays suitable for early cancer detection. Our new cfDNA assay presented here focuses on early breast cancer detection without the need for any prior information on tumor-specific genetic alterations. Because telomere crisis is present in early-stage breast tumors including DCIS we hypothesize that the tumor-derived cfDNA containing shorter telomeres is released and circulates in the bloodstream, thereby enabling us to quantitate telomeric cfDNA abnormality in plasma of women with breast cancer.

While mammography remains the most commonly used method for the screening and early detection of breast cancer, this procedure is often limited by a high medical cost, high false-positive rate necessitating additional testing and leading to patient anxiety, as well as false-negative results which may lead to delay of diagnosis especially for younger women who has dense breast tissues [25–28]. Biomarkers such as cancer antigen 15-3 (CA 15-3) and carcinoembryonic antigen (CEA) have been widely studied in order to monitor disease progression or response to therapy in patients post-diagnosed with breast cancer [29–31], however at this point they have unsuccessfully improved patient outcomes. Thus, there is a critical need for new diagnostic tests for breast cancer, especially those associated with early tumor development. Here, we introduce our newly developed quantitative telomeric cfDNA qPCR assay for measuring the levels of telomeric cfDNA in the plasma. Using this assay, we show a clear relationship between early breast cancer development and quantitative changes in plasma telomeric cfDNA. Our method potentially provides a cost-effective, time-efficient, and convenient blood test for early detection of breast cancer.

## RESULTS

### Measurement methodology of telomeric cell-free DNA in plasma

First, we performed a methodological evaluation to determine an appropriate cell-free DNA (cfDNA) extraction method, including sodium iodide (NaI) method [32] and QIAamp DNA blood kit. We found no apparent differences in total cfDNA yield between healthy control group and cancer group isolated by NaI method (Supplementary Fig. S1). In contrast, QIAamp blood kit showed a slightly but significantly lower yield of total cfDNA in healthy group compared to cancer group. In addition, the cfDNA in healthy group extracted using the Qiagen column showed relatively lower yield than those using the NaI method. To eliminate the potential bias that a certain fraction of cfDNA might be lost during extraction with a silica-based membrane utilized in the QIAamp method (e.g., DNA fragments which are smaller and more difficult to extract [33]), we employed NaI method for isolating cfDNA from each plasma sample for this study. After extracting cfDNA, we carefully measured the cfDNA concentration by a picogreen binding assay to ensure that each cfDNA concentration was at least 10 pg/μL, because this concentration provides the minimum amount of input to maintain the accuracy of the qPCR assay and produce consistent results. No significant correlation was detected between the cfDNA yield and age of the patients (data not shown).

**Figure 1:**
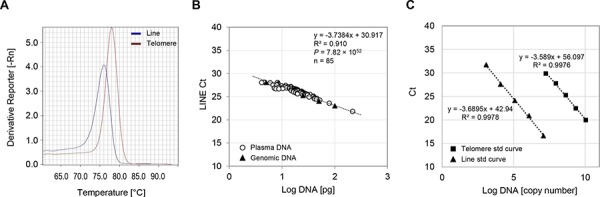
Methodological validation of the telomeric cfDNA qPCR assay **A.** Representative melting curves of single product amplifications using telc/telg and line121F/line121R primers. PCR was performed under 40 cycles of amplification of human plasma cfDNA. -Rn, the negative first-derivative of the normalized fluorescence generated by the reporter during PCR amplification. **B.** Amplification of internal reference sequence reflects input DNA amount of both plasma cfDNA and genomic DNA. Concentrations of experimental plasma cfDNAs (circles) as well as a serially diluted female genomic DNA samples (triangles) were determined using the picogreen assay. After qPCR, the amount of input DNA was plotted logarithmically against the Ct value of the reference primers. **C.** Standard curves of plasmid DNAs for calculating T/R copy ratio. Serially diluted plasmid DNA with known copy numbers of the telomeric or LINE sequences were plotted after the PCR. The amount of plasmid DNA standard used was optimized so that all plasma samples were detected within the linear range of the curves.

Plasma cfDNA is thought to be fragmented and not chromosomal in nature, thereby its DNA ploidy is likely abnormal [34]. Therefore, we expected that single copy genes (such as GAPDH, β-globin) are not suitable for qPCR-based cfDNA assays as internal references. In addition, it is often difficult to amplify single copy genes in ultra-trace amount of circulating cfDNA. To overcome these concerns, we designed four reference primers (see Materials and Methods, Supplementary Table S1) within the Short INterspersed Element (SINE) or Long INterspersed Element (LINE) nucleotide sequences, because approximately 13% and 17% of human genome consists of these non-coding repetitive SINE and LINE elements, respectively [35]. After screening, we chose one primer set (line121F/line121R) designed within the LINE sequences which produced a single 121-bp amplicon (Fig. 1A), whereas others had either multi-amplicons or a gentle peak under our PCR condition; therefore, they were not suitable for quantifying the total amount of input DNA. Most importantly, threshold cycle (Ct) values of the new primer set (line121F/line121R) showed a linear correlation with cfDNA concentrations (by picogreen binding assay), indicating that this reference primer pair accurately reflects the amount of input DNA with minimal sample-to-sample variations (*P* = 7.82 × 10^−52^) (Fig. 1B). To conduct quantitative analyses of the amount of telomeric cfDNA, we generated DNA standard curves using plasmid DNAs with insert sequences of known copy numbers of either telomere or LINE nucleotide sequences (Fig. 1C). The copy number of telomere (T) was determined relative to the copy number of the LINE reference sequence (R) for each cfDNA sample, and expressed as T/R copy ratios. A primer pair for amplifying telomeric sequences was originally designed by Cawthon and produces a single 79-bp amplicon (Fig. 1A) [36]. Throughout all experiments, we included an invariant endogenous control (female diploid genomic DNA) as well as standard plasmid DNAs in each PCR plate to monitor PCR efficiency and maintain accuracy in sample quantitation.

### Plasma telomeric cfDNA amount is inversely associated with age in healthy women

**Figure 2:**
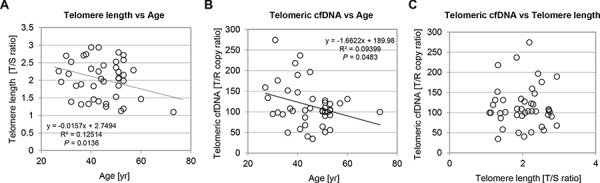
Chronological age has impact on both leukocyte telomere length and plasma telomeric cfDNA level in the control healthy group **A–B.** Relationships between age and leukocyte telomere length (A) or their paired plasma telomeric cfDNA levels (B) Each data from healthy control subjects (*n* = 42) was plotted with age at time of donation. *P*-value of the linear regression was calculated by GraphPad Prism version 5.00. **C.** No strong association between plasma telomeric cfDNA levels (T/R copy ratio) and lymphocyte telomere length (T/S ratio). All plasma T/R results were plotted against their paired lymphocyte telomere length.

It has been well established that average telomere length in peripheral blood leukocyte shortens with increasing age by 17 − 50 bp per year using Southern blotting or Cawthon's qPCR assay [37, 38]. Therefore, we were interested in measuring leukocyte telomere length using our samples from healthy women population (*n* = 42, 27 ≤ age ≤ 73). Both single copy gene primer sets, albumin (ALB) and β-globin (HBG) indicated high reproducibility under our PCR condition (R^2^ = 0.914, *P* < 0.001) (Supplementary Fig. S2A). Consistent with published reports, we confirmed that the healthy women group in this study was also presented with a downward association between leukocyte telomere length and age (R^2^ = 0.125, *P* = 0.0136) (Fig. 2A). Next, to address a question whether age impacts telomeric cfDNA, we examined the distribution of telomeric cfDNA level using plasma samples from the same healthy women population. Interestingly, plasma telomeric cfDNA level was also inversely associated with age with variation (*P* = 0.0483), suggesting that telomeric cfDNA is likely derived from somatic tissues or cells in which telomere length is influenced by age (Fig. 2B). However, there was no association between plasma telomeric cfDNA and leukocyte telomere length in our healthy sample, suggesting that leukocyte DNA is not a major source of cfDNA production (Fig. 2C).

### Level of telomeric cfDNA in plasma from breast cancer patients

**Figure 3:**
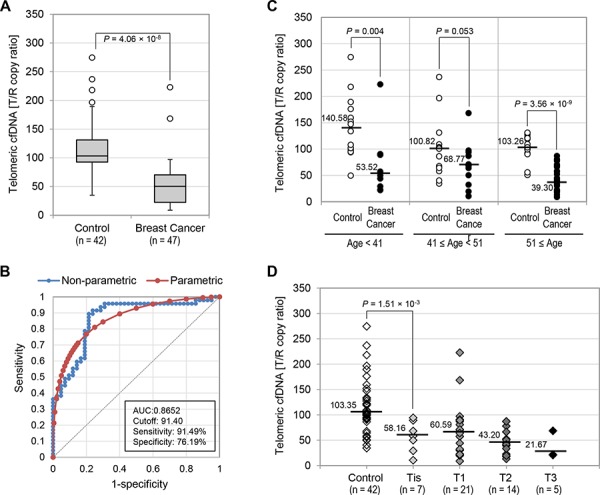
Telomeric cfDNA levels in breast cancer patients were significantly lower than those from controls **A.** Box plot for plasma T/R copy ratios measured in the control and breast cancer groups. **B.** ROC curves for the accuracy of T/R copy ratios in distinguishing breast cancer patients from healthy individuals. Both non-parametric (blue) and fitted parametric curves (red) were shown. **C.** Breast cancer T/R copy ratio abnormality in different age groups. T/R copy ratios were compared between the control group and the breast cancer group in three age categories (≤ 40, 41 – 50, and 51 ≤). **D.** T/R copy ratio comparison in control and breast cancer cases categorized by tumor size. Median T/R copy ratio in each category was shown.

Using the telomeric cfDNA qPCR method described above, we compared telomeric cfDNA levels between two groups, namely healthy individuals (*n* = 42) and a breast cancer group (*n* = 47). The baseline of demographic characteristics was show in Table 1. To avoid potential factors which may change cfDNA levels, we investigated plasma samples from breast cancer patients collected at the time of surgery, prior to any chemo-, radio- or hormone therapies. Overall, the telomeric cfDNA level was significantly lower in the breast cancer group compared to the healthy group. The median telomeric cfDNA as T/R copy ratio in the control and breast cancer group was 103.35 and 50.30, respectively (*P* = 4.06 × 10^−8^) (Fig. 3A). We also determined the sensitivity and specificity of this new assay by construction of Receiver Operating Characteristic (ROC) curves (Fig. 3B). Telomeric cfDNA showed the diagnostic accuracy with an area under the ROC curve (AUC) of 0.8652 (95% Confidence Interval: 0.7867–0.9438, *P* < 0.0001), which is a measure of how well a parameter can distinguish between two groups. At the cutoff value of 91.40 (T/R copy ratio) for telomeric cfDNA, the optimal sensitivity and specificity were 91.49% and 75.19% respectively. Moreover, the positive and negative predictive values (PPV and NPV) were 82.35% and 86.84%, which represents the percentage of patients with a positive and negative test of those individuals who actually have the disease and no disease, respectively. Since we found that telomeric cfDNA decreased with age in the healthy population (Fig. 2A), we divided the results into subsets by age (Fig. 3C). Overall, unlike the control healthy group, there was no correlation between telomeric cfDNA level and age in the breast cancer group. This observation is in agreement with previous findings that telomere length in breast tumor tissues was not associated with age [12], suggesting that a substantial fraction of cfDNA in the plasma of breast cancer patients could be of tumor origin. In the women younger than 41 and older than 51, the T/R cutoff value of 91.40 (T/R copy ratio) readily distinguished healthy from breast cancer patients. However, in the group aged between 41 and 50, telomeric cfDNA level was not able to distinguish breast cancer patient from healthy controls (*P* = 0.053), although we detected a tendency of lower T/R copy ratio in the breast cancer group (Fig. 3C). This statistical insignificance could be due to the small sample size. Since this slight difference mainly resulted from high variation of telomeric cfDNA level in healthy individuals, we speculate that hormonal changes or imbalance at the premenopausal stage may have contributed to this variation.

**Table 1:** Characteristics of study groups

	Healthy individuals	Breast cancer patients	*P*-value
**Total number**	42	47	—
**Age, years**	44.8 ± 9.5	51.0 ± 11.5	0.007
**≤40**	*n* = 14	*n* = 10	—
**41–50**	*n* = 13	*n* = 14	—
**51≤**	*n* = 15	*n* = 23	—
**Sex**	Female	Female	—
**Ethnicity**	Caucasian	Caucasian	—
**Height, cm**	167.3 ± 5.6	164.4 ± 8.8	0.07 (N.S.*)
**Weight, kg**	62.8 ± 6.0	77.3 ± 18.0	<5 × 10^−6^
**BMI**	22.4 ± 1.9	28.5 ± 6.0	<5 × 10^−7^

Values expressed with a plus/minus sign are means ± S.D.

* N.S., Not significant.

Several reports suggest that cfDNA levels are associated with the degree of tumor differentiation, size, or disease stage [39–41]. Thus, we categorized the breast cancer population according to the size of the primary tumor ranged from T_is_ to T_3_. The clinical characteristics of cancer patients were shown in Supplementary Table S2. The T_is_ stage defines carcinoma *in situ* and all 7 cases from the T_is_ stage were DCIS in this study. Notably, our results showed that telomeric cfDNA levels clearly discriminated the DCIS group from the control group (58.16 *vs.* 103.35, *P* = 1.51 × 10^−3^) (Fig. 3D). This finding provides evidence that our newly developed telomeric cfDNA qPCR assay has great promise to capture women at the pre-malignant stage. However, we were not able to determine whether an association exists between telomeric cfDNA levels and tumor size, although there is a tendency of lower T/R copy ratio with increasing tumor size. This is likely due to current limited sample size. The breast cancer population was also categorized according to their lymph node involvement (Supplementary Fig. S3A). Again we did not detect an association between telomeric cfDNA level and lymph node invasiveness, pointing out that our assay detects aberrant telomeric cfDNA reduction even at the N_0_ stage before any lymph node metastasis. Moreover, it is unlikely that adiposity is associated with telomeric cfDNA levels because the telomeric cfDNA amount in breast cancer patients is consistently low regardless of Body Mass Index (BMI) (Supplementary Fig. S3B).

### Level of plasma centromeric cfDNA in both healthy and breast cancer women

**Figure 4:**
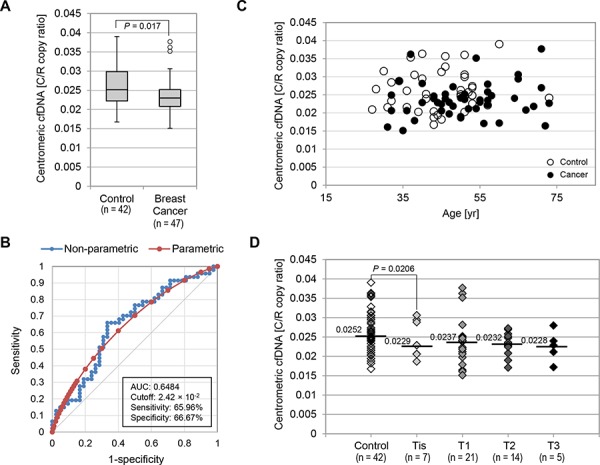
Centromeric cfDNA levels were not highly valuable as a marker for breast cancer detection **A.** Box plot for plasma C/R copy ratios measured in the control and breast cancer groups. **B.** ROC curves for the accuracy of C/R copy ratios in distinguishing breast cancer patients from healthy individuals. Both non-parametric (blue) and fitted parametric curves (red) were shown. **C.** No strong association between centromeric cfDNA levels (C/R copy ratio) and age at time of donation. Open circles, healthy control group; Filled circles, breast cancer cases. **D.** C/R copy ratio comparison in control and breast cancer cases categorized by tumor size. Median C/R copy ratio in each category was shown.

To determine whether tumor-driven cfDNA was abnormal only in amounts of telomeric sequences or amounts of other repetitive sequences, we investigated centromeric cfDNA levels using the same plasma samples described above. Indeed, there have been several observations of centromere instability, such as centromeric fissions found in human cancer [42, 43]. Pan-centromeric primers were designed within alpha-satellite DNA and can amplify as a single product from at least 15 chromosomes *in silico* (Supplementary Fig. S4A). Overall, the median centromeric cfDNA levels in the control group was 2.52 × 10^−2^ as C/R copy ratio, and the median centromeric cfDNA level in the breast cancer group was 2.31 × 10^−2^ as C/R copy ratio (Fig. 4A). Although this difference was statistically significant (*P* = 0.017), an AUC of the ROC curve indicated that the diagnostic value of centromeric cfDNA was not very high (AUC = 0.6484, 95% Confidence Interval: 0.5328 – 0.7640, *P* = 0.0161) (Fig. 4B). At the cutoff value of 2.42 × 10^−2^ (C/R copy ratio) for centromeric cfDNA, the optimal sensitivity and specificity were 65.96% and 66.67% respectively. Moreover, in contrast to telomeric cfDNA levels, centromeric cfDNA levels in the healthy control group were not associated with age (Fig. 4C). When the breast cancer cases were subcategorized by tumor size (T_is_ to T_3_), our results showed that the difference in centromeric cfDNA levels between the DCIS group and the control group was statistically significant (*P* = 0.0206). However, the actual difference in their C/R copy ratios was merely 10% (2.29 × 10^−2^ *vs*. 2.52 × 10^−2^) (Fig. 4D). Thus, these findings suggest that centromeric cfDNA amounts are unlikely to be a desirable marker for cancer detection, although we do not rule out the biological significance of centromeric cfDNA levels in cancer development.

## DISCUSSION

Our present study focused on establishing a new strategy to assess circulating cfDNA for detecting early breast cancer. Since plasma contains ultra-trace amount of circulating cfDNA, roughly around 100 ng per ml of plasma in cancer patients among different laboratories [44, 45], various cfDNA methods often require a certain amplification step to improve enrichment of cfDNA prior to analyzing tumor-specific alterations in cfDNA [46, 47]. The new quantitative real-time PCR method that we applied in this present study has no enrichment step and only requires 50 to 150 pg cfDNA per reaction. Our new method quantitates changes in the amount of repetitive telomeric DNA sequences that are abundant and spread throughout the genome, rather than a single copy sequence per genome.

Circulating cfDNA can be isolated from human plasma, serum and other body fluids, and represents a novel biomarker of interest for cancer diagnosis and monitoring. Increasing studies provide evidence that circulating cfDNA serve as liquid biopsy for monitoring the changes occurring in a patient's cancer in real-time [41, 48]. Initial studies reported that elevated levels of total cfDNA were identified in breast cancer patients compared with healthy controls due to the presence of tumor-driven circulating cfDNA. However, these findings are still inconclusive because large individual variations in cfDNA quantity have been observed among different studies [41, 49, 50]. In this study, we observed no elevated levels of total cfDNA from the cancer group compared to those from the healthy group using the NaI-based extraction method (Supplementary Fig. S1). While the exact mechanism is currently unknown, this could be potentially explained by the following factors: 1) the release of tumor cfDNA might suppress normal somatic cells to release cfDNA, and 2) the systematic capacity of cfDNA clearance/degradation might be higher in breast cancer patients than healthy subjects, thus leading to similar levels of total cfDNA. The integrity of cfDNA has also been investigated by comparing the ratio of apoptotic fragments to non-apoptotic cfDNA fragments [51, 52]. Those studies suggest that apoptotic fragmentations are less abundant in plasma/serum cfDNA from cancer patients compared to that from normal controls. By using qPCR, digital PCR, or sequencing technologies, recent studies reported that *PIK3CA* and/or *TP53* mutations status could be obtained from cfDNA in advanced breast cancer patients before, during and after targeted therapy [20, 48]. However, these studies focused on patients with high tumor burden, and it remains to be evaluated whether similar sensitivity can be achieved in patients at the earlier stages of breast cancer. Moreover, monitoring these two mutations are not sufficient to capture all types of breast cancer; therefore, a panel of cancer-specific gene mutations would have to be assessed.

To the best of our knowledge, our study is the first to report that pre-malignant DCIS patients can be discriminated from healthy individuals using plasma circulating cfDNA. This finding contributes to accumulating evidence that telomere-based crisis in which telomere erosion, genomic instability, and massive cell death are concomitantly increased in tumor lesions, and can be identified during the transition from ductal hyperplasia to DCIS [9, 15]. Although the biology and physiology of cfDNA circulation is not well understood, our data may support the hypothesis that the lower telomeric cfDNA levels in the breast cancer groups (even at the DCIS stage) result from the presence of tumor-derived cfDNA with shortened telomeres in the bloodstream. Further, these findings may suggest that tumor cells with shortened telomeres might be prone to cell death and preferentially provide cfDNA. Notably, we found that telomeric cfDNA levels in cases with advanced breast tumors (stages II – III) was significantly lower than those in cases at earlier stages of breast cancer (stages 0 – I) (*P* = 7.12 × 10^−3^, Supplementary Fig. S5). As telomere length at breast tumor lesions shortens with the development of cancer, this result may strengthen our hypothesis that the decreased telomeric cfDNA levels results from the shortening of telomere length at breast cancer tissues. It is thought that plasma cfDNA from cancer patients is a mixture of tumor-derived and non-tumor-derived DNA. A few studies have estimated a small to modest fraction of tumor-derived cfDNA in the total plasma cfDNA of cancer patients (range 1 – 50%, median ∼ 4%) [48, 53]. Nonetheless, in this present study we demonstrated that plasma telomeric cfDNA levels were significantly lower in breast cancer patients compared to healthy controls while the exact mechanism is not currently understood. Since all previous studies estimating the tumor-derived fraction used only one or two tumor-specific alterations (e.g. specific chromosome arm loss/gain, allelic mutations) to represent the pool of total tumor cfDNA, it is possible that the tumor-derived cfDNA fractions were underestimated and the actual tumor-derived cfDNA may be more abundant than currently thought. Since the main focus of this study is on early detection of cancer rather than cfDNA biology, future characterization of plasma cfDNA (e.g., size distribution, fraction ratio) in cancer patients would lead to better understanding of the cfDNA biology.

In addition, it is speculated that telomeric cfDNA levels might be influenced by interweaving factors such as metabolic regulation, hormone balance, and nuclease activity levels in the bloodstream [54–56]. In fact, our results show that telomeric cfDNA levels in the healthy control group were quite variable (range: 34.42 – 274.51, mean: 115.57, as T/R copy ratio), even though we carefully selected the control group by eliminating any potential confounding factors (see Materials and Methods). Nine out of total 42 controls were detected as false positive, and the majority of these were individuals aged between 41 and 50 (6 of 9 cases). While not definitive, we suspect that a phase of menstrual cycle and/or menstruation status might alter normal telomeric cfDNA levels. In the breast cancer group, our telomeric cfDNA data detected a statistical outlier (as false negative) in two cases which are ER^+^/PR^+^/HER2− and normal BMI from Stage IA without any family history. One possible explanation of these false negative results could be involvement of ALT (alternative lengthening mechanisms of telomeres) pathway in which tumors often have heterogeneous telomere length with relatively longer telomeres [57]. Indeed, the ALT phenotype has been reported in ∼5% of breast epithelial malignancies [58].

Currently telomere length measurement has been utilizing peripheral leukocyte DNA in large cohorts, especially in the scope of estimating cancer risk and mortality. However, results from these studies remain conflicting rather than conclusive. For instance, several case-control studies showed that short leukocyte telomere length was associated with increased risk of breast cancer [59, 60], while others indicated contradictory [61, 62] or insignificant associations [63, 64]. The inconsistent conclusions of these epidemiologic studies are perhaps, in part, explained by the lack of standardized method and analysis between laboratories. Additionally, leukocyte telomere length might not be directly associated with breast epithelial carcinogenesis, because leukocyte telomere length is also influenced by other factors such as smoking, adiposity, exercise, oxidative stress, ultraviolet irradiation, and social and economic status [65, 66]. Therefore, further investigation is critical to understanding the usefulness of leukocyte telomere length as a surrogate marker of cancer risk and mortality. It would also be interesting to further investigate whether our telomere cfDNA qPCR assay can be applied for early detection of not only breast cancer, but other types of cancer as well. If the telomere cfDNA aberration is a universal phenotype in various types of carcinogenesis, this new assay has potential as cancer risk assessment or screening test in other cancer types. It is also important to examine non-cancerous patients with benign disease to determine whether telomeric cfDNA abnormality is correlated specifically with breast carcinogenesis.

In summary, this study highlights the quantitative abnormality of plasma telomeric cfDNA observed in patients with pre-malignant DCIS. The exciting results presented a call for future studies testing the assay's potential for clinical utility. Our approach of measuring the amount of telomeric cfDNA in plasma could serve as a liquid biopsy, which would be useful for improving early cancer detection, either by itself or in combination with other diagnostic tools.

## MATERIALS AND METHODS

### Specimens

The Indiana University Institutional Review Board approved the use of human plasma samples. About 1 mL of frozen plasma samples of breast cancer patients (*n* = 47, 32 ≤ age ≤ 73) and control healthy women (*n* = 42, 27 ≤ age ≤ 73) were obtained through the IU Simon Cancer Center Tissue Procurement and Distribution Core and the Susan G. Komen Tissue bank, respectively. All samples were collected in accordance with standard operating procedures described in the facilities' website and with donor's written informed consent. All healthy samples were obtained from Caucasian women with no family history, no current tobacco nor hormone use, average BMI, and lifetime Gail score < 20%. All breast cancer patient samples were from women who underwent surgery without combination therapies to eliminate potential confounding factors. Blood from cancer patients was obtained at the time of surgery. The demographical and clinical information was shown in Table 1 and Supplementary Table S2.

### Plasma cell-free DNA extraction

Frozen plasma samples were thawed on ice prior to cfDNA isolation and centrifuged for 3 min at ≥ 11,000 × *g* in order to remove residual cells, cell debris, and particulate matter. 150 μL of aliquoted supernatant was used for each cfDNA extraction using a Sodium Iodide (NaI)-based method [32]. Briefly, 150 μL plasma was mixed with equal amount of a 2 × Enzyme reaction solution and proteinase K (Roche) was added to a final concentration of 1 mg/ml. The mixture was incubated at 56°C for 30 minutes, and then combined with 1.5 volume of 7.6 M sodium iodide solution, 1 μL of glycogen (20 mg/ml stock), and 1 volume of isopropanol. After vortexing, the samples were incubated for 30 min at room temperature with agitation. Precipitated cfDNA was centrifuged and washed with 40% w/v isopropanol and then 70% ethanol. Finally, DNA pellet was air-dried and dissolved in 50 μL of TE buffer. For comparison of cfDNA extraction methods, plasma samples were also extracted using the QIAamp DNA Blood Mini kit (Qiagen) according to the manufacturer's instructions with a starting plasma volume of 300 μL and elution of 50 μL in buffer AE.

### Measurement of total plasma DNA concentration

Plasma cfDNA concentrations after extraction were measured using Quant-iT ^TM^ PicoGreen dsDNA Assay Kit (Life Technologies) according to the manufacturer's instruction. A female genomic DNA (cat# G152A, Promega) was diluted to 50 pg/μL and used to generate the low-range standard curve in each plate. Fluorescence intensity was measured with a Synergy 2 Multi-Mode Reader at emission wavelength of 520 nm and excitation wavelength of 480 nm.

### Quantitative PCR-based cfDNA assays

Real-time quantitative PCR (qPCR) were performed on ABI 7500 Real-Time PCR system (Applied Biosystems). The final volume of PCR reaction was 15 μL using 1× SYBR Select (Applied Biosystems) including primers and cfDNA as template. cfDNA was added in each primer set using the following ratio; telomere: centromere: LINE = 5: 1: 1. Therefore, if 150 pg cfDNA was used for telomere primers, 30 pg was used for both centromere and LINE primers. telg and telc primers were used for amplification of the telomere signal (T), which generate a 79 bp product (final concentrations 900 nM each) [36]. The primer sequences were as follows: 5′-ACA CTA AGG TTT GGG TTT GGG TTT GGG TTT GGG TTA GTG T-3′ for telg, and 5′-TGT TAG GTA TCC CTA TCC CTA TCC CTA TCC CTA TCC CTA ACA-3′ for telc. The other primer sequences were used for the pan-centromere signal, cen96F, 5′-TTC ATC TCA CAG AGT TGA ACC TTT CCT TTG-3′, and cen96R, 5′-GGC CTC AAA GTG TAC CAA ATA TCC ACT TG-3′ (final concentrations 400 nM each). For the reference sequence (R), line121F, 5′-GGA TTA AGA AAA TGT GGC ACA TAT ACA CCA TGG- 3′ and line121R, 5′-GAT AGT TTA CTG AGA ATG ATG GTT TCC AAT TTC AT-3′ (final concentrations 250 nM each). The thermal cycling profile is Stage 1 for 10 min at 95°C; Stage 2 for 2 cycles of 15 s at 94°C, 15 s at 49°C; and Stage 3 for 40 cycles of 10 s at 94°C, 10 s at 62°C, 10 s at 74°C.

### Analysis of telomeric and centromeric cfDNA qPCR assay

qPCR results were analyzed by generating plasmid DNA standard curves. Therefore, the relative quantitation is based on T/R or C/R copy ratios of each copy number from the standard curves. Five concentrations of a plasmid DNA sample were prepared with known copies of the telomere, centromere, and LINE (Long INterspersed Element) nucleotide sequences by serial dilution (Fig. 1C and Supplementary Fig. S4B). To monitor and compensate for inter-plate variations in PCR efficiency, each plate included standard plasmid DNAs as well as female genomic DNA (Promega). All experimental DNA samples were repeated at least three times in triplicate. All samples have a standard deviation of less than 0.5 for the threshold cycle (Ct) values. Ct values and melting curve analysis were generated by the 7500 software v2.3. The coefficients of variation (CVs) for the Ct values of telomere, centromere, and LINE primers were 1.5%, 2.2%, and 1.0%, respectively. Melting curve analysis was performed on every run to verify specificity and identity of the PCR products. The telomere amount (T) or centromere amount (C) were presented as T/R or C/R copy ratios where T/R = (telomere copy number) / (Line copy number), and C/R = (centromere copy number) / (Line copy number). The final quantity reported result is the average of three independent experiments.

### Control plasmid DNA preparation for cfDNA assays

The plasmid DNAs containing known sequences of the pan-centromeric or LINE DNA was constructed by ligating the PCR product of the pan-centromere or LINE primers to the pGEM-T easy vector (Promega) or the PCR 2.1 TOPO vector (Life Technologies), respectively. A plasmid containing [TTAGGG]_11_ sequences was obtained from Dr. David Gilley (Indiana University). All plasmid DNAs were diluted in PCR grade water. Copy number calculation was performed as described previously [67]. Plasmids were linearized using proper enzymes before use.

### Telomere length qPCR

Paired leukocyte DNAs along with plasma from healthy women were obtained as a lyophilized power after the Komen Tissue Bank was extracted from whole blood samples using the Flex Star automated system with the AGFStar Fresh WB Extraction Kit (Autogen, cat# AGKT-WB-640). Telomere length qPCR was performed ABI 7500 Real-Time PCR system and analyzed as described previously [36, 68]. The telomere primers (telg and telc, final concentrations 900 nM) were used for telomere signal (T). As internal reference (single copy gene signal = ‘S’), two primer sets within albumin and β-globin genes were used (final concentrations 500 nM each). The β-globin (HBG) primers were described previously [69]. The albumin (ALB) primer sequences were 5′-TTG AAT TTC TGC TCT CCT GCC TGT T-3′ and 5′-GTC ACT TAC TGG CGT TTT CTC ATG C-3′. To calculate the T/S ratios, each DNA standard curve was generated using a pooled female genomic DNA (Promega, G152A) (Supplementary Fig. S2B).

### Statistical analysis

The differences in the distribution of T/R or C/R ratios between breast cancer cases and controls, as well as the final cfDNA yield between the NaI and Qiagen method were compared using the Student's *t*-test, and Pearson's correlation analysis was performed for correlation evaluations. A *P* value of less than 0.05 (two-tailed) was considered significant. Receiver operating characteristic curves (ROCs) were used to evaluate the diagnostic performance of T/R and C/R copy ratios. The non-parametric (empirical) ROC curve, as well as the area under the curve (AUC), standard error, 95% confidence interval (CI), and *P* value were generated using the GraphPad Prism version 5.00 (GraphPad Software, San Diego, California USA). The fitted, parametric ROC curve and statistical analysis were generated and performed by the Web-based Calculator for ROC curves (Java translated by John Eng, Department of Radiology and Radiological Science, Johns Hopkins University School of Medicine) based on the original Fortran program LABROC4 by Charles Metz & colleagues (Department of Radiology, University of Chicago).

## SUPPLEMENTARY FIGURES AND TABLES


