# Supplementary material for: Aberrant reduction of telomere repetitive sequences in plasma cell-free DNA for early breast cancer detection
Source: Oncotarget. 2015 Aug 24;6(30):29795–807. doi: 10.18632/oncotarget.5083 (PMC4745763; doi:10.18632/oncotarget.5083)
Supplement: Supplementary file 1 [file oncotarget-06-29795-s001.pdf]

SUPPLEMENTARY FIGURES AND TABLES

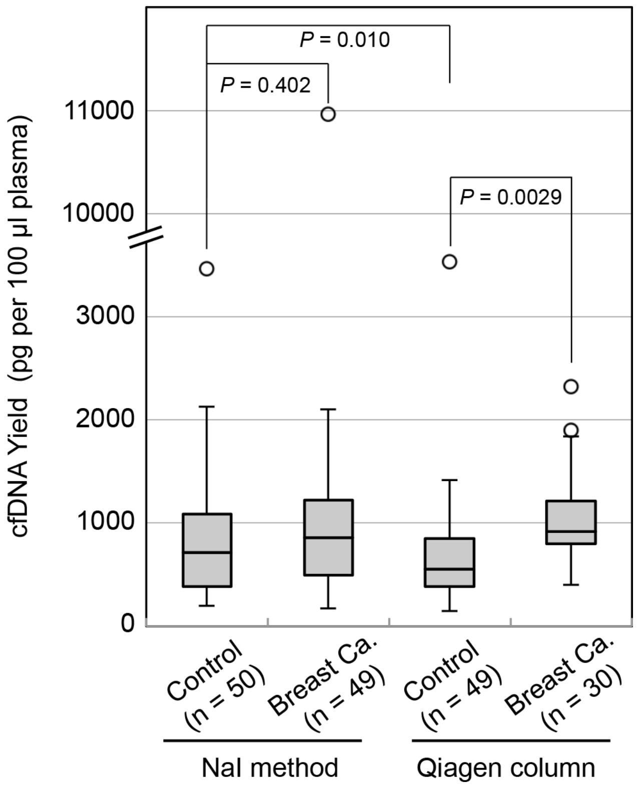

Supplementary Figure S1: Plasma cfDNA yield varies by different extraction methods. Plasma cfDNA concentration from both the control and breast cancer groups was measured by the picogreen binding assay after extraction using the NaI method and the QIAamp DNA Blood Mini column. Box plot showing final cfDNA yield as pg DNA in every 100 µL plasma. *P* value between the control and breast cancer cases was shown for both methods.

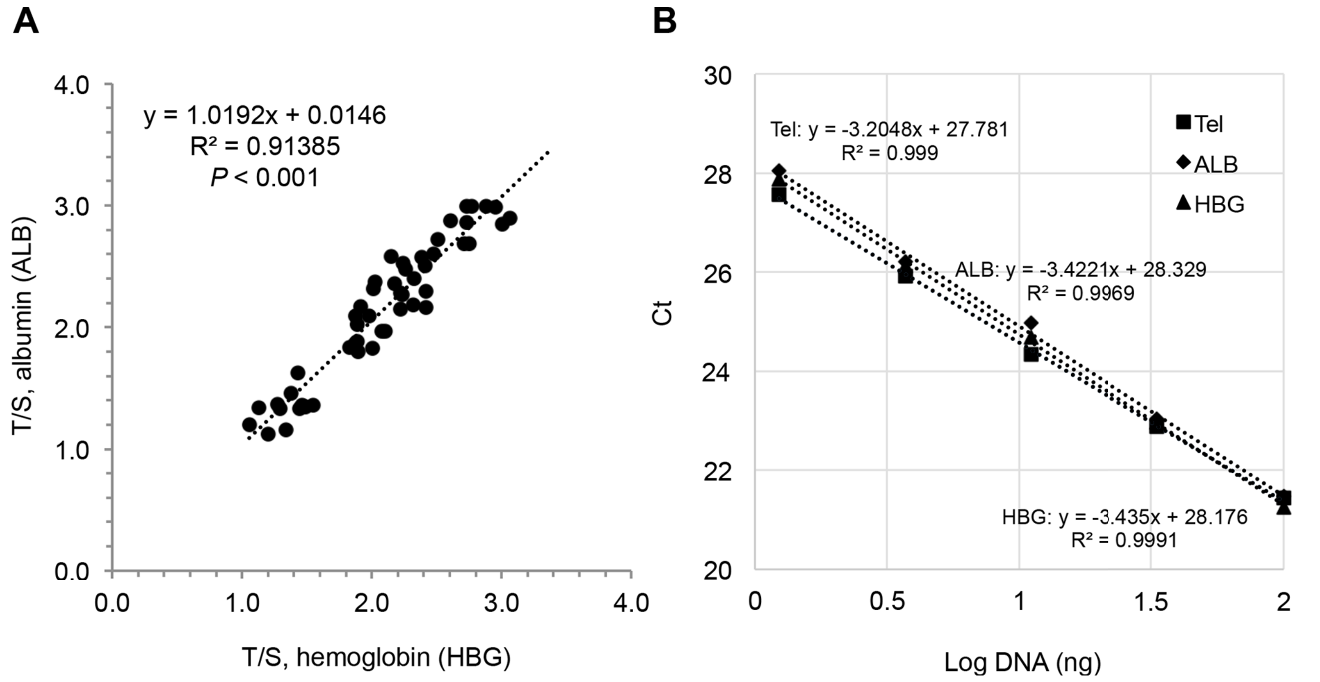

Supplementary Figure S2: Leukocyte telomere length qPCR validation. A. Correlation between T/S ratios obtained with albumin (ALB) versus hemoglobin (HBG) as the internal reference gene. **B.** Standard curves for calculating T/S ratio. A female diploid genomic DNA were 3-fold serially diluted (from 100 ng to 1.23 ng) and used to generate the standard curves. The amount of DNA standard used was optimized to cover all experimental DNA samples. Squares, data for telomere primers; Diamond: data for albumin primers; Triangle: data for hemoglobin primers.

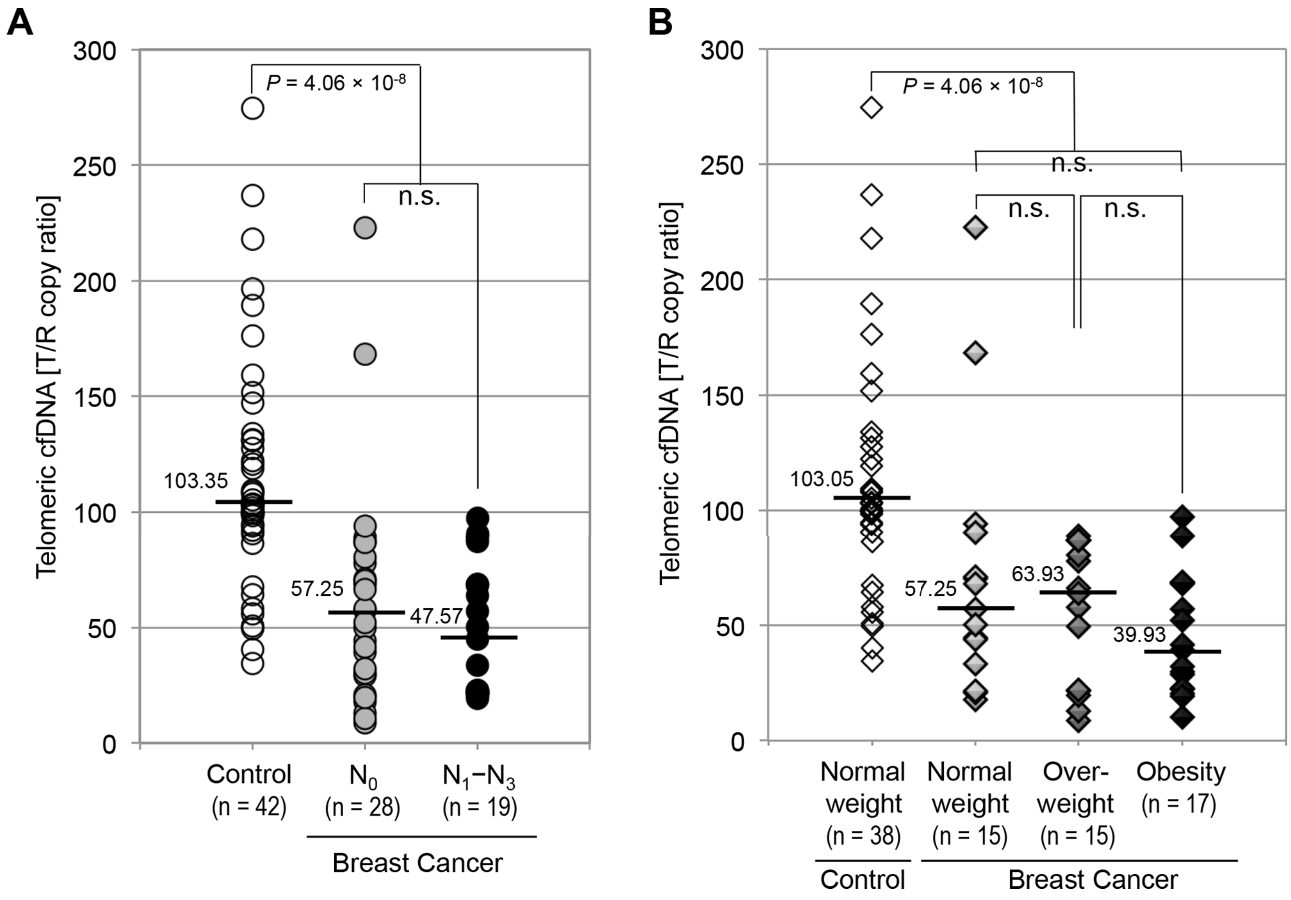

Supplementary Figure S3: Plasma telomeric cfDNA levels in the breast cancer group were consistently low regardless of lymph node invasiveness and degree of adiposity. A. Patients with non-invasive tumor were detected using telomeric cfDNA qPCR assay. N_0, cases without cancer cells in the lymph node: N_1 – N_3, cases with lymph node invasion. **B.** Degree of adiposity was assigned based on the Body Mass Index (BMI). Normal weight, 18.5 ≤ BMI ≤ 24.9; Overweight, 25 ≤ BMI ≤ 29.9; Obesity, 30 < BMI. Median T/R copy ratio in each category was shown in each graph. N.S., *P* value was not significant.

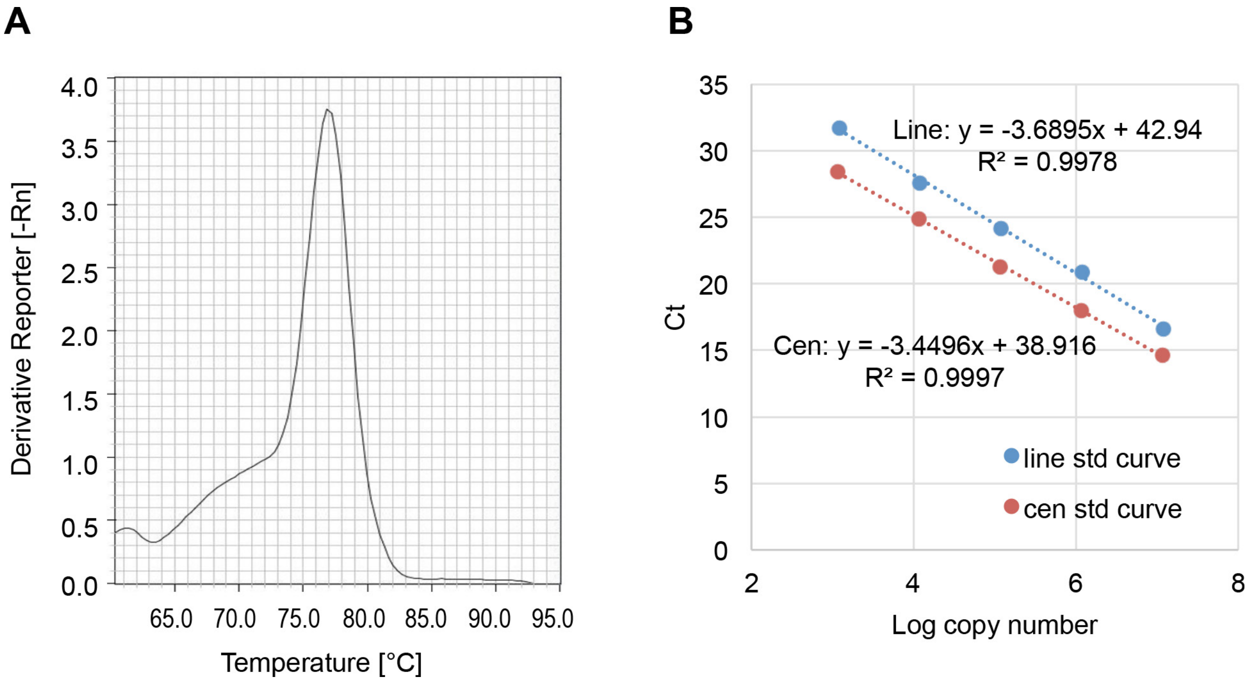

Supplementary Figure S4: Centromeric cfDNA qPCR assay. **A.** Melting curve shows a single amplicon using the centromere primers after 40 cycles of PCR. –Rn, the negative first-derivative of the normalized fluorescence generated by the reporter during PCR amplification. **B.** Standard curves for calculating C/R copy ratio. Plasmids with known copy numbers of the centromere or LINE sequences were serially diluted. DNA concentrations of the plasmid standards were measured with the picogreen fluorescence assay and then converted to copy number based on their molecular weight. The amount of plasmid DNA standard used was optimized so that all plasma samples were detected within the linear range of the curves.

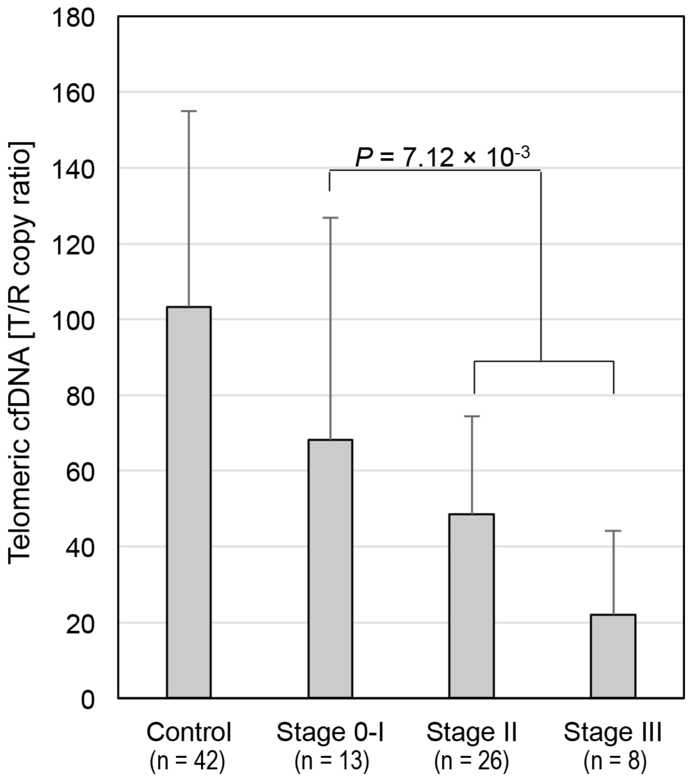

Supplementary Figure S5: Telomeric cfDNA levels were decreased with progression of breast cancer. T/R copy ratio comparison in control and breast cancer cases categorized by stage. Each bar represents median T/R copy ratio in the corresponding category ± standard deviation.

Supplementary Table S1: Primer sequences that were screened but not chosen

Target	Primer Sequence
Alu115	Forward: 5′-CCT GAG GTC AGG AGT TCG AGA CCA GCC TG-3′
	Reverse: 5′-CTC CCG AGT AGC TGG GAT TAC AGG CG-3′
Line79	Forward: 5′-AGG GAC ATG GAT GAA ATT GG-3′
	Reverse: 5′-TGA GAA TAT GCG GTG TTT GG-3′
Line87	Forward: 5′-GAA TCT CAC TCA AAG CCG CTC AAC TAC ATG-3′
	Reverse: 5′-TCT GCC TTC ATT TCG TTA TGT ACC CAG-3′

Supplementary Table S2: Characteristics of tumors

	No. of patients (%)
**Total number**	47 (100%)
**Histologic type**	
Ductal carcinoma	42 (89.4%)
Lobular carcinoma	2 (4.2%)
Ductal and lobular	3 (6.4%)
**Hormone receptor status***	
$ER^+/PR^+/HER2^-$ (Luminal A)	29 (61.7%)
$ER^+/PR^+/HER2^+$ (Luminal B)	2 (4.2%)
$ER^+/PR^+/HER2^\pm$	6 (12.8.%)
$ER^-/PR^-/HER2^+$ (HER2 type)	1 (2.1%)
$ER^-/PR^-/HER2^-$(Triple negative)	3 (6.4%)
N.A.****	6 (12.8%)
**Tumor stage****	
Tis	7 (14.9%)
T_1	21 (44.7%)
T_2	14 (29.8%)
T_3	5 (10.6%)
**Lymph node*****	
N_0	29 (61.7%)
N_1	12 (25.5%)
N_2	1 (2.1%)
N_3	5 (10.6%)

* ER/PR test results are interpreted according to the American Society of Clinical Oncology/College of American Pathologists (CAP) guidelines 2014. For ER/PR status, immunohistochemistry staining (IHC) of > 1% is considered positive; For HER2, IHC 3+ = positive; IHC 2+ = borderline (±); IHC 1+ or 0 = negative. ER, estrogen receptor; PR, progesterone receptor; HER2, human epidermal growth factor receptor 2.
** Tis, Carcinoma *in situ*. All 7 cases were DCIS; T_1, Tumor <= 2 cm across; T_2: Tumor is more than 2 cm but less than 5 cm across; T_3: Tumor > 5 cm across.
*** N_0, no cancer cells in the lymph node; N_1, metastasized to 1–3 axillary lymph node(s) including internal mammary lymph nodes; N_2, metastasized to 4–9 lymph nodes under the arm, or enlarged the internal mammary lymph nodes; N_3, either metastasized to lymph nodes below the collarbone, in the armpit and behind the breast bone, or above the collarbone.
**** N.A., Information is not available.
